# Using place-based characteristics to inform FDA tobacco sales inspections: results from a multilevel propensity score model

**DOI:** 10.1136/tobaccocontrol-2021-056742

**Published:** 2021-10-25

**Authors:** Hongying Dai, Lisa Henriksen, Zheng Xu, Nirosha Rathnayake

**Affiliations:** 1 Department of Biostatistics, University of Nebraska Medical Center, Omaha, Nebraska, USA; 2 Stanford Prevention Research Center, Stanford UniversitySchool of Medicine, Stanford, California, USA; 3 Department of Mathematics and Statistics, Wright State University, Dayton, Ohio, USA

**Keywords:** public policy, surveillance and monitoring, environment

## Abstract

**Background:**

Conducting routine inspections for compliance with age-of-sale laws is essential to reducing underage access to tobacco. We seek to develop a multilevel propensity score model (PSM) to predict retail violation of sales to minors (RVSM).

**Methods:**

The Food and Drug Administration compliance check of tobacco retailers with minor-involved inspections from 2015 to 2019 (n=683 741) was linked with multilevel data for demographics and policies. Generalised estimating equation was used to develop the PSM using 2015–2016 data to predict the 2017 RVSM. The prediction accuracy of the PSM was validated by contrasting PSM deciles against 2018–2019 actual violation data.

**Results:**

In 2017, 44.3% of 26 150 zip codes with ≥1 tobacco retailer had 0 FDA underage sales inspections, 11.0% had 1 inspection, 13.5% had 2–3, 15.3% had 4–9, and 15.9% had 10 or more. The likelihood of having an RVSM in 2017 was higher in zip codes with a lower number of inspections (adjusted OR (aOR)=0.988, 95% CI (0.987 to 0.990)) and penalties (aOR=0.97, 95% CI (0.95 to 0.99)) and a higher number of violations (aOR=1.07, 95% CI (1.06 to 1.08)) in the previous 2 years. Urbanicity, socioeconomic status, smoking prevalence and tobacco control policies at multilevels also predicted retail violations. Prediction accuracy was validated with zip codes with the highest 10% of the PSM 3.4 times more likely to have retail violations in 2019 than zip codes in the bottom decile.

**Conclusion:**

The multilevel PSM predicts the RVSM with a good rank order of retail violations. The model-based approach can be used to identify hot spots of retail violations and improve the sampling plan for future inspections.

Tobacco use by youth and young adults can harm brain development and increases the likelihood of nicotine addiction.^1 2^ Evidence suggests that routine monitoring and enforcement of age-of-sale restrictions are essential to reduce youth tobacco use.^1^ Before federal law increased the minimum legal sales age for tobacco from 18 to 21 years in December 2019,^3^ the per cent of US middle and high school students (ages 9–17 years) who reported buying tobacco declined from 15.6% in 2016 to 11.4% in 2018.^4^ However, the per cent of youth who reported being refused a tobacco sale remained constant, at approximately 25%. In addition, nearly 60% of US middle or high school students reported that it was ‘easy’ or ‘somewhat easy’ to buy tobacco products in stores.^5^ In California, which requires tobacco retail licensing and adopted Tobacco 21 in 2017, less than 30% of purchasers (ages 19–20 years) were refused purchase of cigarettes or e-cigarettes because of their age.^6^

Under the Family Smoking Prevention and Tobacco Control Act, the US Food and Drug Administration (FDA) works through state contractors to inspect tobacco retailers for marketing violations and underage sales.^7^ Although the FDA advises states to consider the factors associated with high risk of regulatory violations (eg, areas with high rates of youth smoking prevalence, easy access to cigarettes and minority communities),^8^ states are not required to use probability-based sampling strategies, and the inspection protocol is inconsistent across states.^9^ Past studies have found significant variations in the number of compliance inspections per capita and retail violation of sale to minors (RVSM) across states.^10 11^ For instance, the violation rate ranged from 1.4% (Montana) to 29.4% (New Hampshire) in the 2015 FDA compliance inspections.^11^ Empirical data are critically needed to inform probability-based inspection sampling design, which can leverage prior inspection results and other contextual factors to adaptively optimise inspection frequency and further provide tobacco retail education to the regions with a high propensity of RVSM. Simulation experiments have shown that the probability-based sampling design would help strengthen the FDA’s compliance and enforcement programme, save money and resources by increasing inspection efficiency, and emphasise inspections in areas where they are needed most to reduce health disparities.^9^

According to recent studies of FDA inspections, retail violations for sales to minors were more likely to occur in neighbourhoods of lower socioeconomic status (SES) with a higher percentage of minority residents and weaker tobacco control policies.^11^ However, these findings, based on association studies, could lead to inspections targeting neighbourhoods with specific profiles (such as a high prevalence of African Americans) instead of identifying areas with an increased likelihood of underage sales violation. Furthermore, prior regression modelling did not account for previous retail inspection, violation and penalty as the explanatory variables, which have been shown to be significant factors associated with reduced alcohol sales to underage buyers.^12 13^ A propensity score model (PSM) that aims to predict the likelihood of future RVSM by incorporating these multilevel predictors may identify areas with a higher risk of underage violations and thus inform the FDA of potentially optimal sampling strategies for retail compliance inspections.

To fill this critical gap, we harnessed multilevel and longitudinal data that link 2015–2019 FDA compliance inspections with zip code-level SES and other contextual factors, such as county-level smoking prevalence, state and local adoption of Tobacco 21, and other state tobacco control policies. We then developed and validated a multilevel PSM to predict whether the inspection resulted in an RVSM and further demonstrated the opportunity to identify areas with a high risk of violations in applying the PSM for future compliance inspections.

## Data and method

### Tobacco Retail Compliance Checks

FDA’s undercover buy inspections involve the use of a minor under the supervision of the inspectors to evaluate whether a retailer sells tobacco products to individuals who are under 18 years old.^14^ The inspections also assess whether a retailer requests photo identification to verify age for individuals who attempt to purchase tobacco products and appear to be 27 years or younger.^15^ FDA’s public database of inspection results includes inspected retailers (ie, retailer name, address, city, zip code), inspection decisions (‘no violations observed’, ‘warning letter’, ‘civil money penalty’ and ‘no-tobacco-sale order’), whether a minor was involved in the inspection (yes/no), whether sale to minor was found in the inspection (yes/no/not applicable) and decision date.^14^ Warning letters are issued to tobacco retailers following their first compliance check violation. Civil money penalties are issued after a second compliance check violation, and no-tobacco-sale orders are issued after ‘repeated violations’, which are defined as at least five violations of particular requirements over a 36-month period.^16^ We obtained the inspection data from 1 January 2015 to 31 December 2019 (n=781 055) and included only the 683 741 undercover buy inspections indicated as ‘minor involved’ in 50 states and the District of Columbia.

Each inspection result was classified as a binary variable: no violation (coded as 0) and violation (coded as 1, including warning letter, civil money penalty and no-tobacco-sale order). We also classified the inspection results based on whether there was a penalty (coded as 1, including civil money penalty or no-tobacco-sale order) or not (coded as 0, including no violation or warning letter). We aggregated inspection results (ie, number of inspections, number of violations and number of penalties) from each zip code over 2 years to serve as predictors (eg, 2015 and 2016 inspections for the 2017 PSM development, 2016 and 2017 inspections for the 2018 validation).

### Demographic correlates

Data from 5-year estimates (2014–2018) of the American Community Survey (ACS) were used to investigate demographic correlates of RVSM. We selected zip code-level variables that are known to be associated with retail violations of tobacco sales to minors,^11 13 17^ such as total population, age (ie, per cent of persons 10–17 years old, 18–20 years old and 21–24 years old); race and ethnicity (ie, per cent of non-Hispanic white, per cent of non-Hispanic black, per cent of Hispanic, per cent of non-Hispanic Asian and percentage of American Indian/Alaskan Native); education (ie, per cent of persons aged 25 years or above with bachelor’s degree or higher) and income (ie, per cent of persons living in poverty). Regarding urbanicity, the Rural–Urban Commuting Area (RUCA) codes were used to classify each zip code as metropolitan or non-metropolitan, which combined micropolitan, small town and rural.^11 18^ The RUCA system derives the classification based on the commuting pattern with the 2010 decennial census and the 2006–2010 ACS data.^19^

### Tobacco retailer data and smoking prevalence

Data were obtained from ReferenceUSA^20^ to identify 358 070 likely tobacco retailers as of December 2016 (see table 1 footnote). This data source has been used in multiple studies.^21 22^ We calculated the number of tobacco retailers per zip code, and restricted statistical modelling to zip codes with ≥1 tobacco retailer. Data for the 2016 county-level smoking prevalence were obtained from the County Health Rankings & Roadmaps program.^23^

**Table 1:** Sample characteristics of zip codes, by number of FDA compliance inspections in 2017 (129 911 inspections clustered in n=26 131 zip codes)

Zip codes with various levels of compliance inspections in 2017*						
Number of inspections	**0**	**1**	**2–3**	**4–9**	**10+**	**Total**
Number of zip codes	11 572	2874	3516	4009	4160	26 131
% of total	44.3	11.0	13.5	15.3	15.9	100.0
**Zip code-level characteristics**						
FDA inspections and violations						
Number of inspections, 2017 (mean)†	0.0	1.0	2.4	6.0	22.8	5.0
Number of violations, 2017 (mean)†	0.0	0.1	0.4	0.9	3.3	0.7
Number of penalties, 2017 (mean)†	0.0	0.0	0.1	0.3	1.0	0.2
Number of tobacco retailers (mean)‡	9.0	7.6	9.3	15.7	32.7	13.7
Metropolitan (%)§	47.6	44.7	48.2	59.9	73.9	53.5
**Zip code-level demographics**						
Population (mean)¶	8792	6999	8206	13 661	26 731	12 200
Race/ethnicity						
Caucasian (%)¶	75.0	82.9	81.7	76.4	67.2	75.7
African American (%)¶	6.9	7.0	7.4	9.6	13.7	8.5
Hispanic (%)¶	11.3	5.8	6.4	8.7	12.6	9.8
Asian (%)¶	2.4	1.4	1.6	2.5	3.6	2.4
American Indian (%)¶	2.2	0.9	1.1	0.7	0.5	1.4
Age (years)						
10–17 (%)¶	10.3	10.4	10.4	10.4	10.1	10.3
18–20 (%)¶	3.8	3.6	3.6	3.8	4.2	3.8
21–24 (%)¶	4.8	4.5	4.5	5.0	5.8	4.9
Education and income						
Bachelor’s degree or higher (%)¶	18.7	18.5	19.1	21.9	23.5	20.0
Poverty (%)¶	14.9	14.2	13.7	14.4	16.1	14.8
**County-level characteristics**						
Adult smoking prevalence (%)	16.6	17.6	17.3	17.0	16.6	16.9
Zip code-level Tobacco 21 policies**						
No (%)	86.6	95.8	95.9	95.0	91.5	90.9
Local Tobacco 21 (%)	2.8	2.3	2.7	3.4	5.9	3.3
State Tobacco 21 (%)	10.6	1.9	1.4	1.6	2.6	5.8
**State-level Tobacco Control policies**						
Cigarette tax** (mean)	$1.4	$1.7	$1.7	$1.7	$1.7	$1.6
Tobacco retail licensing**						
E-cigarettes or other tobacco (%)	10.5	22.1	23.7	20.6	19.2	16.5
Tobacco other than e-cigarettes (%)	63.6	57.3	55.7	56.6	57.5	59.8
No license required (%)	25.9	20.6	20.6	22.8	23.3	23.7

*Zip codes are divided into five groups based on the number of tobacco retail inspections involving minors.

†2017 data with inspections involving minors from FDA tobacco retail database were averaged at the zip code level.

‡Tobacco retail data obtained from ReferenceUSA and averaged at the zip code level for all businesses that likely sold tobacco products as of December 2016 using North American Industry Classification System codes, such as 445 110 (supermarkets and grocery stores), 445 120 (convenience stores or food marts), 445 310 (beer, wine and liquor stores), 446 110 (pharmacy and drug stores), 447 110 (gasoline stations with convenience stores), 447 190 (other gasoline stations), 452 990 (discount stores and general stores), 452 910 (warehouse clubs and superstores) and 453 991 (tobacco stores). We further excluded national chain retailers that do not sell tobacco products (eg, Target, CVS, Whole Foods, Dollar Tree) and removed duplicate records.

§Each zip code was coded as 1 for metropolitan or 0 for non-metropolitan (ie, micropolitan, small town and rural) using the Rural–Urban Commuting Area codes.

¶Zip code-level demographic information was obtained from the American Community Survey, 2014–2018.

**As of December 2016.

FDA, Food and Drug Administration.

### Time-variant tobacco control policy data

Data for Tobacco 21 ordinances at both local and state levels were obtained from the University of Missouri Tobacco Control Research Center,^24^ including location (county or city), state and effective dates. We geocoded and mapped each US zip code to jurisdiction or county. We then categorised the adoption status of Tobacco 21 laws as non-Tobacco 21, local Tobacco 21 policies and state Tobacco 21 policies as a time-variant variable in December 2015–2019 based on the policy effective dates.

Data for state cigarette tax and tobacco retailer licensing requirements across all 50 states and the District of Columbia for each year from 2014 to 2019 were obtained from the Centers for Disease Control and Prevention’s State Tobacco Activities Tracking and Evaluation System.^25^ Tobacco retailer requirement was classified as three mutually exclusive groups: (1) license required to sell e-cigarettes and other tobacco products, (2) license required to sell tobacco but not required to sell e-cigarettes, and (3) no license required.

### Statistical analysis

#### Data management and descriptive analysis

**Figure 1:**
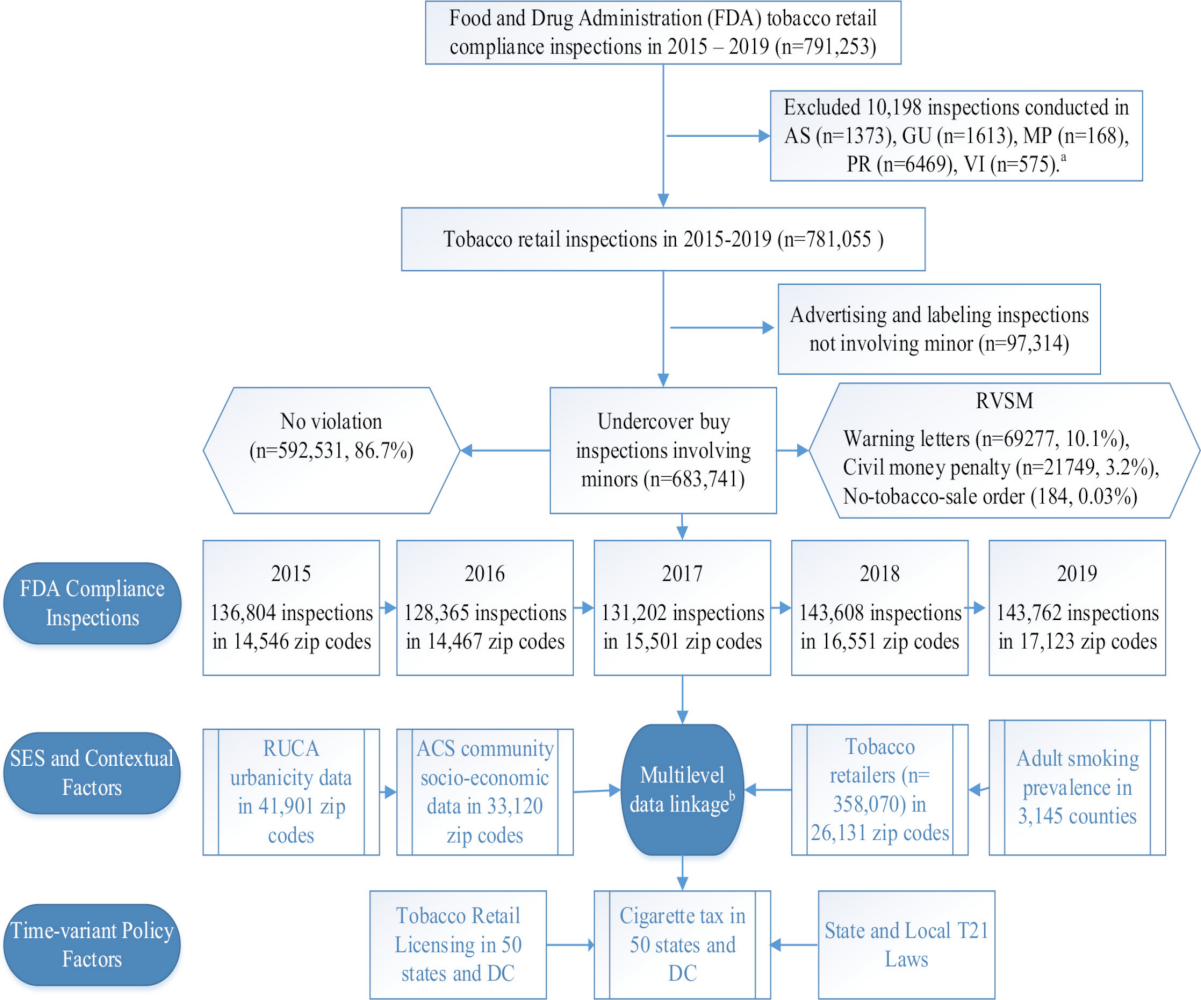
Flow chart for sample selection in FDA tobacco retail compliance inspections and multilevel data linkage with community characteristics and tobacco control policies. a: the final analysis is limited to 50 states and the District of Columbia (DC), so inspections in US territories were removed. These territories include American Samoa (AS), Guam (GU), Northern Marianas Islands (MP), Puerto Rico (PR) and US Virgin Islands (VI). b: multilevel factors, including zip code-level past inspection results and SES along with other contextual factors, such as county-level smoking prevalence, state and local adoption of Tobacco 21, and other state tobacco control policies using geographic information system. These multilevel factors were treated as covariates (explanatory variables) in the PSM development and validation. ACS, American Community Survey; PSM, propensity score model; RUCA, Rural–Urban Commuting Area; RVSM, retail violation of sales to minors; SES, socioeconomic status; T21, Tobacco 21.

The multilevel data from different sources were linked at the zip code, county and state levels using ArcGIS V.10.5 (see figure 1 for data linkage). For descriptive purposes, zip codes were divided into five groups based on the number of tobacco retail inspections involving minors (0, 1, 2–3, 4–9 and 10+) in 2017. The neighbourhood characteristics were summarised overall and by these inspection frequency categories (table 1).

#### PSM development

The PSM for RVSM (binary dependent variable: 1—yes, vs 0—no at each inspection) is constructed as follows:

(1) $$\ln\left(\frac{P_{izcst}}{1-P_{izcst}}\right)=\beta_{0}+\beta_{1}X_{zt}+\beta_{2}M_{ct}+\beta_{3}N_{st}+e_{z\left(s\right)}+e_{i}$$

where all predictive variables are listed in the first column of table 2. Here $P_{izcst}$ is the probability of RVSM for the $i^{th}$ inspection in the $z^{th}$ zip code, $c^{th}$ county and $s^{th}$ state at the $t^{th}$ time. The vectors $X_{zt}$ , $M_{ct}$ , and $N_{st}$ represent predictive variables at the zip code level, county level, and state level, respectively. Generalised estimating equation (SAS ‘Genmod’ procedure^26^) was performed to model random effects ( e ) using repeated measures. We performed varying nested models to select a parsimonious random effect structure that accounts for spatial autocorrelation among geographical units (zip codes nested in the state) without making the model too complex. OR from the bivariate regression and adjusted OR (aOR) from the multivariable regression analysis were reported in table 2.

**Table 2:** Multilevel propensity score model (PSM) to predict a retail violation of sales to minors (RVSM) in 2017 (129 911 inspections clustered in n=26 131 zip codes)*

Predictive variables	OR (95% CI)	P value	aOR (95% CI)	Adjusted p value
**Zip code-level inspections and retailers**				
Number of inspections 2015–2016†	1.00 (1.00 to 1.00)	0.6658	0.988 (0.987 to 0.99)	<0.0001
Number of violations 2015–2016†	1.02 (1.02 to 1.03)	<0.0001	1.07 (1.06 to 1.08)	<0.0001
Number of penalties 2015–2016†	1.06 (1.05 to 1.08)	<0.0001	0.97 (0.95 to 0.99)	0.0011
Number of tobacco retailers‡	1.21 (1.09 to 1.35)	0.0007	Not included	
**Zip code-level characteristics**				
Population§	1.00 (1.00 to 1.00)	0.0045	1.00 (1.00 to 1.01)	0.0011
Metropolitan (vs non-metropolitan)	1.22 (1.17 to 1.28)	<0.0001	1.3 (1.22 to 1.38)	<0.0001
Race/ethnicity¶				
Caucasian (%)	0.95 (0.94 to 0.96)	<0.0001	Not included**	
African American (%)	1.08 (1.07 to 1.09)	<0.0001	1.04 (1.03 to 1.06)	<0.0001
Hispanic (%)	1.01 (1.00 to 1.03)	0.1577	0.99 (0.98 to 1.01)	0.5383
Asian (%)	0.90 (0.86 to 0.94)	<0.0001	0.94 (0.89 to 0.98)	0.0101
American Indian (%)	1.19 (1.10 to 1.28)	<0.0001	1.19 (1.10 to 1.29)	<0.0001
Age distribution (years)¶				
10–17 (%)	0.85 (0.79 to 0.93)	0.0002	0.80 (0.73 to 0.88)	<0.0001
18–20 (%)	1.10 (1.03 to 1.18)	0.0062	1.04 (0.95 to 1.15)	0.3729
21–24 (%)	1.15 (1.07 to 1.24)	0.0001	0.99 (0.89 to 1.10)	0.8253
Education and income¶				
Bachelor’s degree or higher (%)	0.96 (0.94 to 0.98)	<0.0001	0.97 (0.95 to 1.00)	0.0188
Poverty (%)	1.14 (1.11 to 1.17)	<0.0001	1.02 (0.98 to 1.07)	0.2504
**County-level characteristics**				
Adult smoking prevalence (%)¶	1.28 (1.20 to 1.36)	<0.0001	1.13 (1.03 to 1.23)	0.0068
**Zip code-level Tobacco 21 policies**††				
No	Reference		Reference	
Local	0.72 (0.64 to 0.81)	<0.0001	0.70 (0.63 to 0.79)	<0.0001
State	0.49 (0.40 to 0.61)	<0.0001	0.50 (0.4 to 0.62)	<0.0001
**State-level characteristics**				
Cigarette tax ($)†††	0.97 (0.95 to 0.99)	0.0011	Not included**	
Tobacco retail licensing††				
E-cigarettes or other tobacco products	0.58 (0.54 to 0.62)	<0.0001	0.66 (0.62 to 0.71)	<0.0001
Tobacco products other than e-cigarettes	0.81 (0.77 to 0.85)	<0.0001	0.83 (0.78 to 0.87)	<0.0001
No license required	Reference		Reference	

*PSM development: multilevel propensity score was developed using generalised estimating equation model, where the dependent variable was RVSM (yes vs no) at each inspection in 2017 and the explanatory variables included all variables listed in the first column as fixed effects along with the random effects shown in equation (1).ORs from univariate analysis and aORs from the multivariable analysis are reported.

†Per 1 unit increase.

‡Per 100 units increase.

§Per 1000 people increase.

¶Per 10% increase.

**Due to multicollinearity with other predictors, Caucasian (%) and state cigarette tax were not included in the multivariable regression.

††As of December 2016.

aOR, adjusted OR.

The propensity score was then calculated as the probability of RVSM at each inspection by applying the corresponding regression coefficient to each predictor with the formula:

(2) $$P_{i}zcst=\frac{exp({\hat{\beta }}_{0}+{\hat{\beta }}_{1}X_{z}t+{\hat{\beta }}_{2}M_{c}t+{\hat{\beta }}_{3}N_{s}t)}{\left(1+exp({\hat{\beta }}_{0}+{\hat{\beta }}_{1}X_{z}t+{\hat{\beta }}_{2}M_{c}t+{\hat{\beta }}_{3}N_{s}t)\right)}$$

where ${\hat{\beta }}_{0}-{\hat{\beta }}_{4}$ are regression coefficients. Since all predictors were at the zip code or higher level, all inspections in the same zip code will receive the same PSM.

#### PSM validation

**Figure 2:**
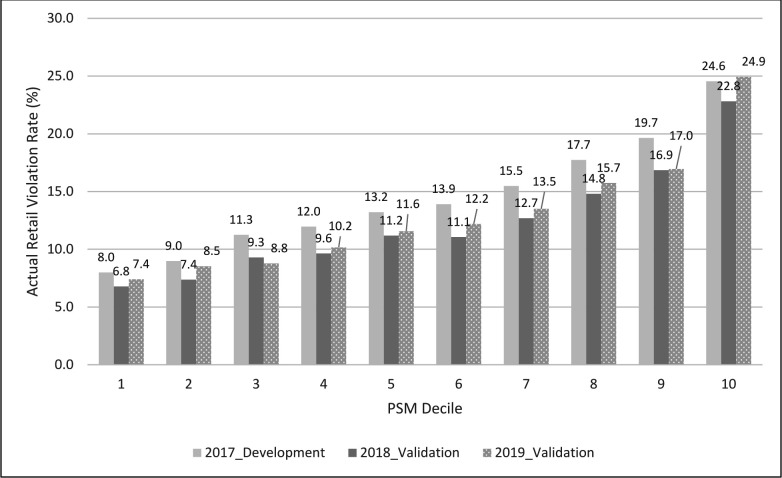
Multilevel propensity score model (PSM) prediction of RVSM at the zip code level (n=26 131 with ≥1 tobacco retailer): 2017^a^ vs 2018^b^ vs 2019^c^. a: 2017 (PSM development): multilevel propensity score was developed where the dependent variable was RVSM (yes vs no) in 2017 and the explanatory variables included the past 2-year (2015–2016) inspection results along with other contextual factors (see equation 1). The propensity score was ranked in the order from the lowest (1, the bottom decile) to the highest (10, the top decile) in the 2017 data. b: 2018 (PSM validation): the developed PSM was prospectively validated using the 2018 FDA data where the dependent variable was RVSM (yes vs no) in 2018 and the explanatory variables included the past 2-year (2016–2017) inspection results along with other contextual factors (see equation 2). The propensity score was ranked in the order from the lowest (1, the bottom decile) to the highest (10, the top decile) in the 2018 data. c: 2019 (PSM validation): the developed PSM was prospectively validated using the 2019 FDA data where the dependent variable was RVSM (yes vs no) in 2019 and the explanatory variables included the past 2-year (2017–2018) inspection results along with other contextual factors (see equation 2). The propensity score was ranked in the order from the lowest (1, the bottom decile) to the highest (10, the top decile) in the 2019 data. FDA, Food and Drug Administration; RVSM, retail violation of sales to minors.

We validated the PSM prediction accuracy between the development sample (2017 inspection data) and two separate validation samples (2018 or 2019 inspection data) based on the following steps: (1) we calculated the predicted PSM score at each inspection in 2017, 2018 or 2019 based on previous 2-year inspection results at the zip code level (2015–2016, 2016–2017 or 2017–2018) and other multilevel contextual factors as predictors (see equation 2); (2) we ranked individual inspection data from the lowest to the highest PSM score (representing the lowest to the highest potential for a violation) at each inspection and then split up the ranked data into 10 equal subsections (ie, deciles); (3) we plotted the actual retail violation rates at each decile (the number of violations/the number of inspections) in each year by the decile of PSM score in figure 2 to illustrate the model performance across the development and validation samples.

In order to demonstrate potential opportunities in applying the PSM score, we further reported the distribution of US zip codes with ≥1 tobacco retailer and the number of tobacco retailers by the PSM score deciles, and the number of FDA compliance inspections in 2017 in table 3. All statistical analyses were performed using SAS V.9.4, and p value of <0.05 was considered statistically significant.

**Table 3:** % of zip codes by decile of PSM and number of FDA compliance inspections (n=26 131 with ≥1 tobacco retailer), 2017

Decile of PSM for RVSM*†	Number of compliance inspections at each zip code, 2017					
	0	1	2–3	4–9	10+	Total
1	12.8	5.0	4.8	6.3	13.8	10.0
2	7.1	12.1	12.9	11.7	12.2	10.0
3	9.3	10.4	11.5	10.8	9.7	10.0
4	9.9	12.3	10.7	10.3	7.9	10.0
5	9.5	11.1	10.8	10.2	9.8	10.0
6	9.9	11.3	10.3	10.6	8.5	10.0
7	10.6	10.5	10.4	10.2	7.5	10.0
8	10.7	10.2	10.4	10.1	7.5	10.0
9	10.1	10.0	9.9	10.2	9.6	10.0
10	10.2	7.0	8.1	9.7	13.4	10.0
Total	100.0	100.0	100.0	100.0	100.0	100.0

*The propensity score was ranked in the order from the lowest (1, the bottom decile) to the highest (10, the top decile) in predicting 2017 RVSM.

†Column (%) is shown in the table.

FDA, Food and Drug Administration; PSM, propensity score model; RVSM, retail violation of sales to minors.

## Results

Of 26 131 zip codes with tobacco retailers in 2017, 44.3% (11 589) had no FDA compliance inspections involving minors, 11.0% (2875) had 1 inspection, 13.5% (3517) had 2–3 inspections, 15.3% (4009) had 4–9 inspections, and 15.9% (4160) had 10 or more inspections. Table 1 presents the sample characteristics of zip codes, grouped by the number of compliance inspections in 2017.

Table 2 presents the unadjusted OR and aOR of risk factors from the multilevel PSM to predict RVSM of each inspection (yes/no) in 2017. In the multivariable analysis, zip codes with a lower number of past 2-year inspections (aOR=0.988; 95% CI (0.987 to 0.990)), a higher number of violations (aOR=1.07; 95% CI (1.06 to 1.08)) and a lower number of penalties (aOR=0.97; 95% CI (0.95 to 0.99)) in 2015–2016 were more likely to have an RVSM in 2017. Metropolitan zip codes were more likely than non-metropolitan counterparts to have an RVSM (aOR=1.30; 95% CI (1.22 to 1.38)). Higher odds of RVSM were found in zip codes with a higher proportion (per 10% increase) of African Americans (aOR=1.04; 95% CI (1.03 to 1.06)) and American Indians/Alaskan Natives (aOR=1.19; 95% CI (1.10 to 1.29)). In contrast, zip codes with a higher proportion of Asians, youth (ages 10–17 years) or residents with higher education were less likely to have an RVSM. County-level smoking prevalence was positively associated with RVSM. Notably, Tobacco 21 policies and state tobacco retail licensing requirements were negatively associated with RVSM. Compared with zip codes without Tobacco 21 policies, those where local policies existed had a lower odds of an RVSM (aOR=0.70; 95% CI (0.63 to 0.79)), and this same pattern was true for state Tobacco 21 policies (aOR=0.50; 95% CI (0.40 to 0.62)). Compared with zip codes in states without tobacco retail licensing, those in states that required licensing for e-cigarettes or other tobacco products (aOR=0.66; 95% CI (0.62 to 0.71)) or requirements for tobacco products other than e-cigarettes (aOR=0.83; 95% CI (0.78 to 0.87)) were less likely to have an RVSM.

The multilevel PSM outcomes from the development sample (2017) and validation samples (2018 or 2019) are presented in figure 2, which plots the observed retail violation rate at each decile of the PSM (=number of violations at each decile/number of inspections at the same decile). Overall, the PSM ranks correspond well with actual outcomes. For instance, the retail violation rate was 24.6% in 2017, 22.8% in 2018 and 24.9% in 2019 with the top decile of predicted PSM, in comparison with 8.0% in 2017, 6.8% in 2018 and 7.4% in 2019 at zip codes with the lowest decile of PSM. The map of predicted propensity scores of RVSM in 2019 among zip codes with tobacco retailer(s) in the USA is presented in online supplemental figure 1.

10.1136/tobaccocontrol-2021-056742.supp1 Supplementary data

Table 3 reports the percentage of zip codes with any tobacco retailers by the PSM decile and the number of FDA compliance inspections in 2017. About 10.2% of zip codes with no compliance inspections in 2017 are in the top PSM decile, 31.0% in the top three deciles and 12.8% in the bottom decile. A similar proportion was observed for the zip codes with 10+ compliance inspections with 13.4% in the top PSM decile, 30.5% in the top three deciles and 13.8% in the bottom decile. Online supplemental table 1 shows the number of tobacco retailers by the PSM score deciles and the number of FDA compliance inspections in 2017. It highlighted that 43 535 tobacco retailers were located in high PSM deciles (eg, ≥7) that were not inspected in 2017.

## Discussion

Given a large number of tobacco retailers (n>350 000) in the USA and the high cost of retail inspections,^16^ development of a probability-based sampling strategy for tobacco retail inspection and education will help identify heterogeneity of sales to minor violations and may reduce disparities in underage access. In this study, we developed a multilevel PSM to predict the likelihood of a federal sales inspection that violated the underage sales laws. In addition, the propensity score was a good predictor of future underage sales violations. In an independent validation sample, we found that zip codes with the top decile of predicted potential violations had 3.3 times higher actual retail violation rates in 2019 versus those in the bottom decile (24.9% vs 7.6%).

To our knowledge, this is the first study to incorporate inspection history, compliance outcomes, and penalties in conjunction with SES and the number of tobacco retailers at the zip code level, smoking prevalence rate at the county level and tobacco control policies at multiple levels to predict future tobacco retail compliance of underage sales laws. Although previous studies have reported that neighbourhood characteristics and tobacco control policies are associated with retail violations of underage sales,^11 13 17^ the current study also found that previous inspection and violation outcomes play an important role in future compliance. Markedly, areas with a larger number of inspections and severe penalties (eg, no-tobacco-sale order) in the previous 2 years were less likely to have a sales violation, while areas with a larger number of violations were more likely to continue to report violations. This is important given prior evidence that FDA is underusing the no-tobacco-sale order: only 1.9% of frequently violating retail locations received that penalty, and the agency could have issued orders sooner.^27^ Results from the current study provide further evidence that FDA should better leverage penalties to deter continued sales violations by tobacco retailers. To the extent that retail violations are concentrated in economically disadvantaged areas and higher smoking prevalence, youth may self-select tobacco retailers known to sell to minors.^11 28^ Thus, random sampling of retail inspection targets may not serve to effectively prevent sales violations in high-risk areas or deter retailers from selling tobacco products to minors. Indeed, we further validated the PSM on an independent dataset to predict violations in 2018 and 2019. The results were largely consistent with the development model, suggesting that the model may be relatively robust to predict future retailer violations.

The current study provides further evidence of the role of strong tobacco control policies in reducing youth access to tobacco products. For instance, state-level Tobacco 21 policies were associated with 50% lower odds of a sales violation in 2019 and local-level Tobacco 21 policies were associated with 30% lower odds of a sales violation. Notably, tobacco retail licensing requirements were associated with a 19%–33% reduction in the odds of a sales to minor violation. However, there were still 13 states without any tobacco retail licensing requirements as of December 2020.^25^ Results of this study suggest that further efforts to implement and enforce comprehensive tobacco policies may improve retail compliance with underage sales laws.

The PSM identified opportunities to improve sampling designs for retail inspections. For instance, 44% of zip codes with a tobacco retailer were not inspected in 2017, over a half of these zip codes are located in the non-metropolitan areas and had an average of 9.0 tobacco retailers at each zip code. The PSM analysis shows that these zip codes were not less risky than other zip codes: 31.0% of non-inspection zip codes were located in the top 30% of the PSM vs 30.5% of those with 10+ compliance inspections in 2017. Furthermore, over 43 000 tobacco retailers with a high propensity score for violations were not inspected in 2017. Since the FDA is spending hundreds of millions of dollars each year in compliance inspection and the Substance Abuse and Mental Health Services requires states to hit a target retail violation rate in order to receive block funding,^16 29^ the propensity score can be useful to identify hot spots of retail violations and to optimise the sampling plan for future compliance inspections.

This study is subject to limitations. First, there might be other confounding factors affecting the retail violations, which are not accounted for in this study. However, this study sought to include a variety of multilevel factors that have been shown to associate with tobacco retail compliance.^11 13 17^ Second, this study did not include tobacco retail inspections conducted by other tobacco prevention programmes, primarily due to the unavailability of data. However, the FDA tobacco retail inspection database provides a large sample size of retail inspection by covering a wide geographical distribution and long inspection period. Third, we did not consider the provisions and strength of local/state Tobacco 21 policies,^30^ local cigarette taxes and local licensing (eg, licensing fees). Furthermore, there is a lack of data about how these policies were enforced at the state or local level, which could also influence the rate of sales violations. Finally, studies have shown that inspection outcomes were related to characteristics of the undercover decoys (eg, appearance, experience).^17^ However, this study treated each inspection and penalty equally in the development of PSM due to the lack of public data to address these characteristics, which should be considered in the allocation of inspection resources in the real-world setting.

## Conclusion

This study developed a multilevel propensity score to predict the retail violations of underage sales to minors, which can be used to identify hot spots of retail violations and improve the sampling plans in the future. This study also adds to the literature by investigating associations between time-variant variables such as previous inspections, violations, penalties, and future inspection and violation outcomes. The data-driven propensity can provide state and local authorities rich information to prioritise regions with compliance disparities and provide resources to reduce tobacco-related disparities due to youth access to tobacco. Although the study focuses on age-of-sale inspections, the finding can be extended to determine priority regions that need tobacco retail training, signage and other intervention programmes.

**What this paper adds**

- Previous research has shown that restricting the supply of tobacco products through age-of-sale restrictions is an essential component in reducing youth smoking.
- Retail violations of underage sales can be predicted with previous inspection results, in conjunction with social-ecological and contextual factors at the community level.
- The propensity score model can be used to identify regions with a high risk of retail violations and improve the sampling plan in future compliance inspections.

## Data Availability

Data are available in a public, open access repository. The data of FDA Compliance Check Inspections of Tobacco Product Retailers can be accessed via https://www.accessdata.fda.gov/scripts/oce/inspections/oce_insp_searching.cfm.
