# Supplementary material for: Using place-based characteristics to inform FDA tobacco sales inspections: results from a multilevel propensity score model
Source: Tob Control. 2021 Oct 25;31(e2):e148–55. doi: 10.1136/tobaccocontrol-2021-056742 (PMC9726945; doi:10.1136/tobaccocontrol-2021-056742)
Supplement: Supplementary data [file tobaccocontrol-2021-056742supp001.pdf]

Appendix Figure 1. Predicted Propensity Score of RVSM over 26,131 zip codes with ≥1 tobacco retails in 2019 [a]

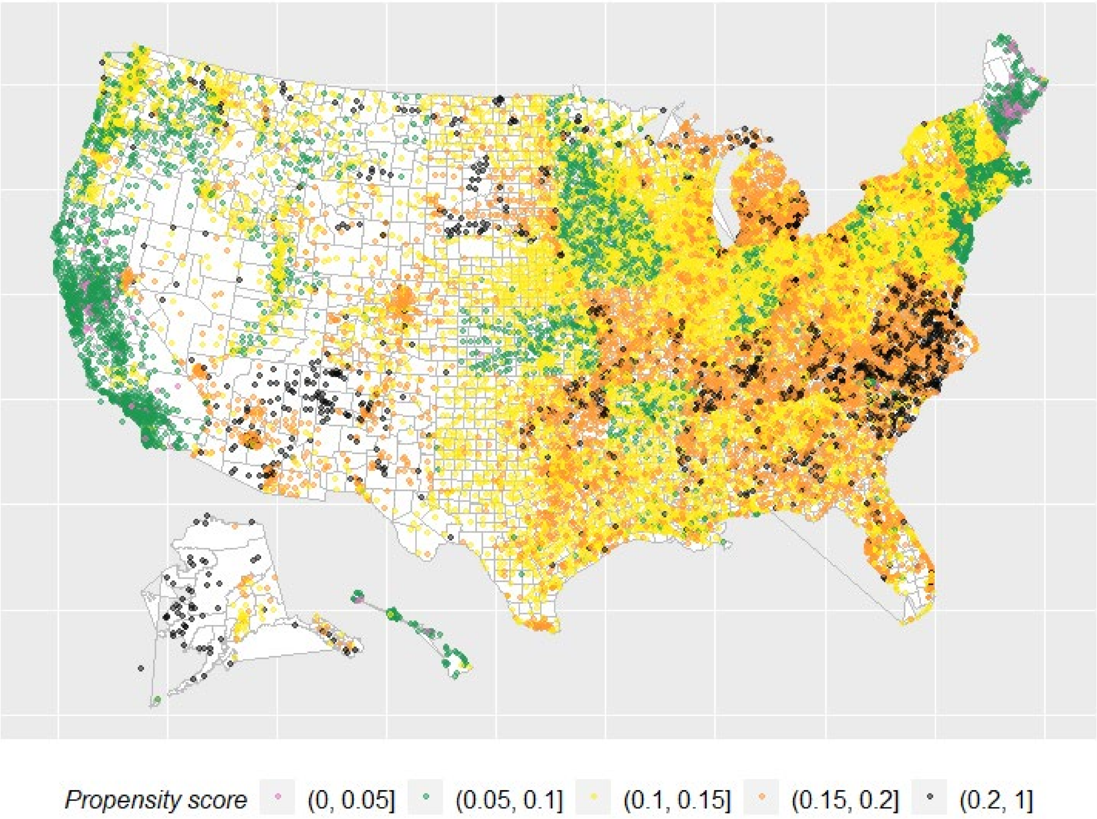

[a.] Each dot stands for the centroid of zip codes with tobacco retailers.

Appendix Table 1. Number of Tobacco Retailers by Decile of PSM and Number of FDA Compliance Inspections (n=26,131 with ≥1 tobacco retailer), 2017

Decile of PSM for RVSM[a]	Number of compliance inspections at each zip code, 2017					
	0	1	2-3	4-9	10+	Total
1	24,809	2,513	3,075	5,481	21,011	56,889
2	5,606	1,166	2,624	4,938	15,408	29,742
3	6,308	1,449	2,704	5,304	12,682	28,447
4	6,468	1,991	2,780	5,770	9,700	26,709
5	7,709	2,196	3,117	5,863	12,368	31,253
6	8,801	2,417	3,374	6,723	10,660	31,975
7	10,227	2,335	3,149	6,494	9,481	31,686
8	10,968	2,678	3,647	7,315	9,428	34,036
9	10,692	2,766	4,333	6,897	13,199	37,887
10	11,648	2,075	3,639	7,898	21,706	46,966
Total	104,549	21,880	32,622	63,020	135,999	355,590

[a] The propensity score was ranked in the order from the lowest (1, the bottom decile) to the highest (10, the top decile) in predicting 2017 RVSM.
